# The discovery of *Candidatus* Nanopusillus phoceensis sheds light on the diversity of the microbiota nanoarchaea

**DOI:** 10.1016/j.isci.2024.109488

**Published:** 2024-03-11

**Authors:** Yasmine Hassani, Gerard Aboudharam, Michel Drancourt, Ghiles Grine

**Affiliations:** 1 Aix-Marseille Université, IRD, MEPHI, IHU Méditerranée Infection, 13005 Marseille, France; 2 IHU Méditerranée Infection, 13005 Marseille, France; 3 Ecole de Médecine Dentaire, Aix-Marseille Université, 13005 Marseille, France

**Keywords:** Biological sciences, Microbiology, Bacteriology, Microbiome

## Abstract

To further assess the spectrum of nanoarchaea in human microbiota, we prospectively searched for nanoarchaea in 110 leftover stool specimens, using the complementary approaches of PCR-sequencing screening, fluorescent *in situ* hybridization, scanning electron microscopy and metagenomics. These investigations yielded a nanoarchaea, *Candidatus* Nanopusillus phoceensis sp. nov., detected in stool samples by specific PCR-based assays. Microscopic observations indicated its close contact with the archaea *Methanobrevibacter smithii*. Genomic sequencing revealed 607,775-bp contig with 24.5% G + C content encoding 30 tRNAs, 3 rRNA genes, and 1,403 coding DNA sequences, of which 719 were assigned to clusters of orthologous groups.

*Ca*. Nanopusillus phoceensis is only the second nanoarchaea to be detected in humans, expanding our knowledge of the repertoire of nanoarchaea associated with the human microbiota and encouraging further research to explore the repertoire of this emerging group of nanomicrobes in clinical samples.

## Highlights

• Discovery of *Candidatus* Nanopusillus phoceensis
• Prospective screening reveals nanoarchaea in 110 stool samples
• Genomic characterization of *Ca.* Nanopusillus phoceensis highlights unique genetic features
• Association between *Ca.* Nanopusillus phoceensis and *Methanobrevibacter smithii*

## Graphical abstract

**Figure:**
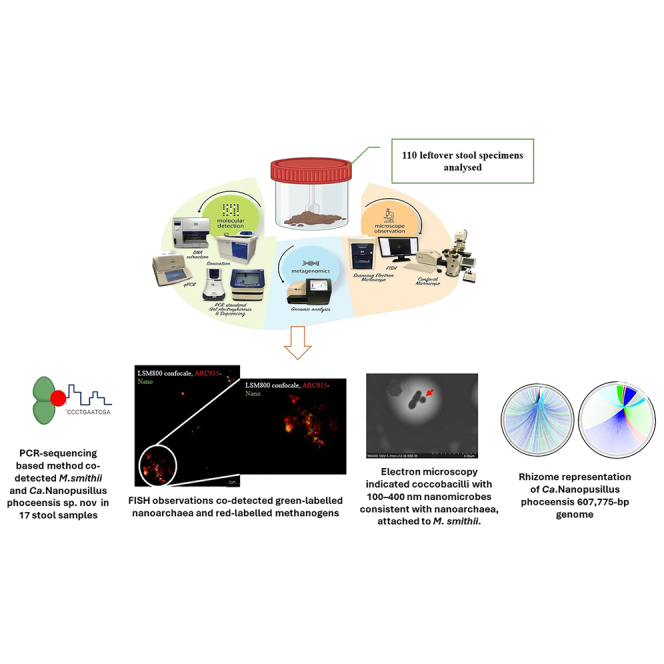

## Introduction

Advances in culture-independent methods, including phylogenetics and genomics including single-cell genomics, has led to the proposal of a candidate microbial monophyletic superphylum, “DPANN,” consisting of five phyla, “Ca. Diapherotrites,” “Ca. Parvarchaeota,” “Ca. Aenigmarchaeota,” “Ca. Nanohaloarchaeota,” and Nanoarchaeota.^1^ The latter phylum currently includes four members which have been detected in some extreme environments^2^^,^^3^^,^^4^^,^^5^ along with *Nanopusillus massiliensis* (*N. massiliensis*), the sole human microbiota-associated representative that we recently discovered and isolated in co-culture with its methanogenic archaea host *Methanobrevibater oralis* (*M. oralis*), from a dental plate specimen.^6^ Although nanoarchaea present heterogeneity on the genomic level,^7^^,^^8^ they share certain common characteristics, featuring 150–400 nm nano-organisms encasing a 400–600-Mgb genome encoding for a reduced metabolic repertoire, and lacking major biosynthetic pathways for the synthesis of amino acids, nucleotides, and lipids. They thus require an obligatory association with another host archaea microorganism.^9^ These recently discovered nanomicrobes, which represent a significant proportion of the microbial world,^9^ are not present in a pure cultivable state, due to the specific characteristics mentioned earlier. Pioneering research into nanoarchaea in human microbiota has led us to invent specific laboratory tools for their detection and isolation, through the co-culture of nanoarchaea in clinical samples,^6^ building on techniques previously described in our laboratory that facilitated the detection of methanogenic archaea in pathological situations, including abscesses,^10^^,^^11^^,^^12^^,^^13^ vaginosis,^14^^,^^15^ urinary tract infection,^16^ and archaeamia.^17^

Nanoarchaea have sparked considerable interest due to their enigmatic presence in the human microbiota. These nano-organisms form symbiotic associations with methanogens species in the oral cavity.^6^ While the precise roles of nanoarchaea in the human microbiota are still being unraveled, their presence holds intriguing implications. They might influence the metabolism and metabolic interactions of their archaeal partners, potentially affecting the production of metabolites like methane, which carries relevance for human health. Moreover, understanding the spectrum of nanoarchaea in the digestive microbiota could have significant health implications, shedding light on their associations with specific health conditions and contributing to our knowledge of disease mechanisms. As we delve deeper into the world of nanoarchaea, their role in maintaining microbiota stability and resilience is also emerging as a fascinating area of study, with potential implications for overall health and microbial ecosystem balance.

To further assess the spectrum of nanoarchaea in the digestive microbiota, we prospectively searched for nanoarchaea in leftover stool specimens, using complementary approaches including PCR-sequencing screening, fluorescent *in situ* hybridization (FISH), scanning electron microscopy, and metagenomics.

## Results

### PCR-sequencing-based investigation of nanoarchaea/methanogens

A total of 110 leftover stool specimens revealed the detection of methanogen DNA in 92 samples (82.6%) (Table S1) in the presence of negative controls, which all remained negative. Further sequencing indicated that 87/92 amplicons (95%) identified *M. smithii*, with 100% 16S rRNA gene sequence similarity; 2/92 stool amplicons (2.17%) exhibited 97.71% sequence similarity with the reference 16S rRNA gene sequence of the *Methanomassiliicoccaceae* archaeon DOK 16S ribosomal RNA gene (accession NCBI: CP047880.1); 1/92 amplicon identified the *Candidatus* Methanomassiliicoccus intestinalis isolate MGYG-HGUT-02160 (accession NCBI: LR698974.1) with 100% sequence similarity; 1/92 amplicon exhibited 100% sequence similarity with the reference 16S rRNA gene sequence of *Methanobrevibacter* sp. AbM23 16S ribosomal RNA gene (accession NCBI: KF697723.1); and 1/92 amplicon exhibited 100% sequence similarity with the reference 16S rRNA gene sequence of *M. oralis*, strain VD9 16S ribosomal RNA gene (accession NCBI: LN898260.1). In addition, nanoarchaea were detected by PCR in 17 leftover stool samples, all of which were also positive for *M. smithii*, whereas negative controls remained negative. In greater detail, sequencing indicated that 9/17 (53%) amplicons exhibited 95% sequence similarity with homologous 30S L12 gene sequence of *N. massiliensis* (accession NCBI: HDO8852.1) and 8/17 (47%) amplicons exhibited 95.44% sequence similarity with homologous 30S L12 gene sequence of *Nanopusillus acidilobi* (*N. acidilobi*) (accession NCBI: CP010514.1).

### Microscopic observation

**Figure 1:**
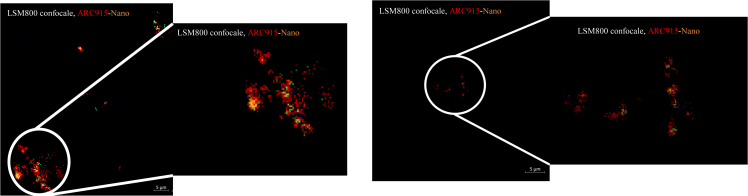
Fluorescent *in situ* hybridization representing the detection of nanaorchaea and methanogens in stools, red ARC 915 and green nanoarchaeota 515mcR2 probes exhibiting organisms with the coccis form stuck to the surface of a methanogen Scale bar, 5 μm.

**Figure 2:**
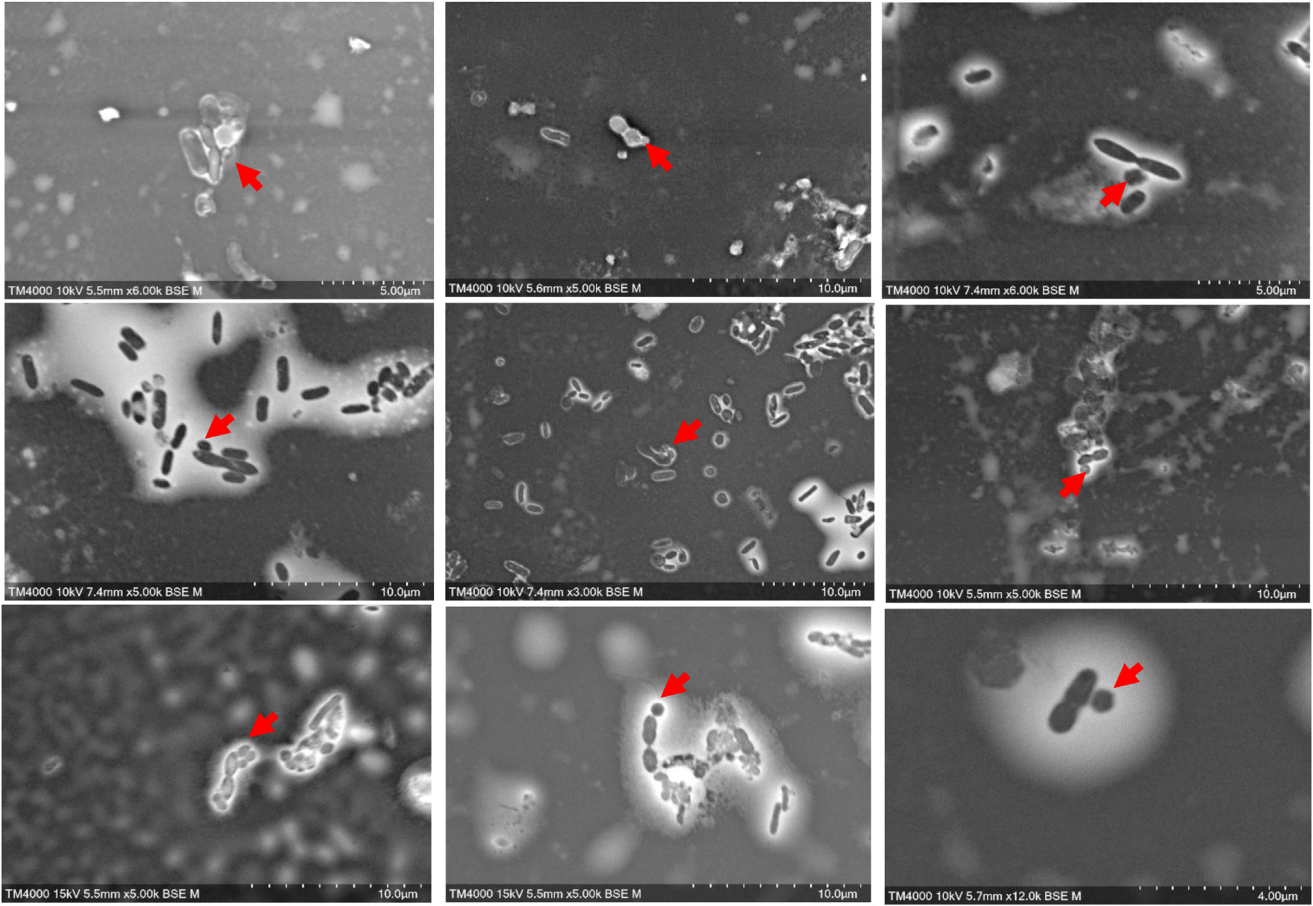
Nanoarchaeota in stools were observed using scanning electron microscopy with TM4000PLus (Hitachi)

FISH observations of the 17 nanoarchaea PCR-positive leftover stool specimens co-detected green-labeled nanoarchaea and red-labeled methanogens in all specimens and the co-localization of the two microbes emitting yellow fluorescence, while sterile PBS-negative controls remained negative. Specifically, FISH revealed diplococcus methanogens suggesting *M. smithii* with attached cocci, the size of which was consistent with that described for nanoarchaea (Figure 1). In addition, electron microscopy indicated coccobacilli in pairs or short chains consistent with *M. smithii*, with 100–400 nm nanomicrobes consistent with nanoarchaea, attached to *M. smithii*. We observed the presence of a thick biofilm, and many cocci ranging in size from 100 to 400 nm attached to the external surface of several diplococci forms evoking the description of *M. smithii* (Figure 2).

While indeed electron microscopy observations relied only on morphology and size compatible with the identification of nanoarchaea at large, FISH provided specificity for the novelty of the identification, as FISH probe is specific for the nanoarchae. At last, PCR results relying on specific primers were comforting progressive results of microscopy.

### Genomic sequencing and description

**Figure 3:**
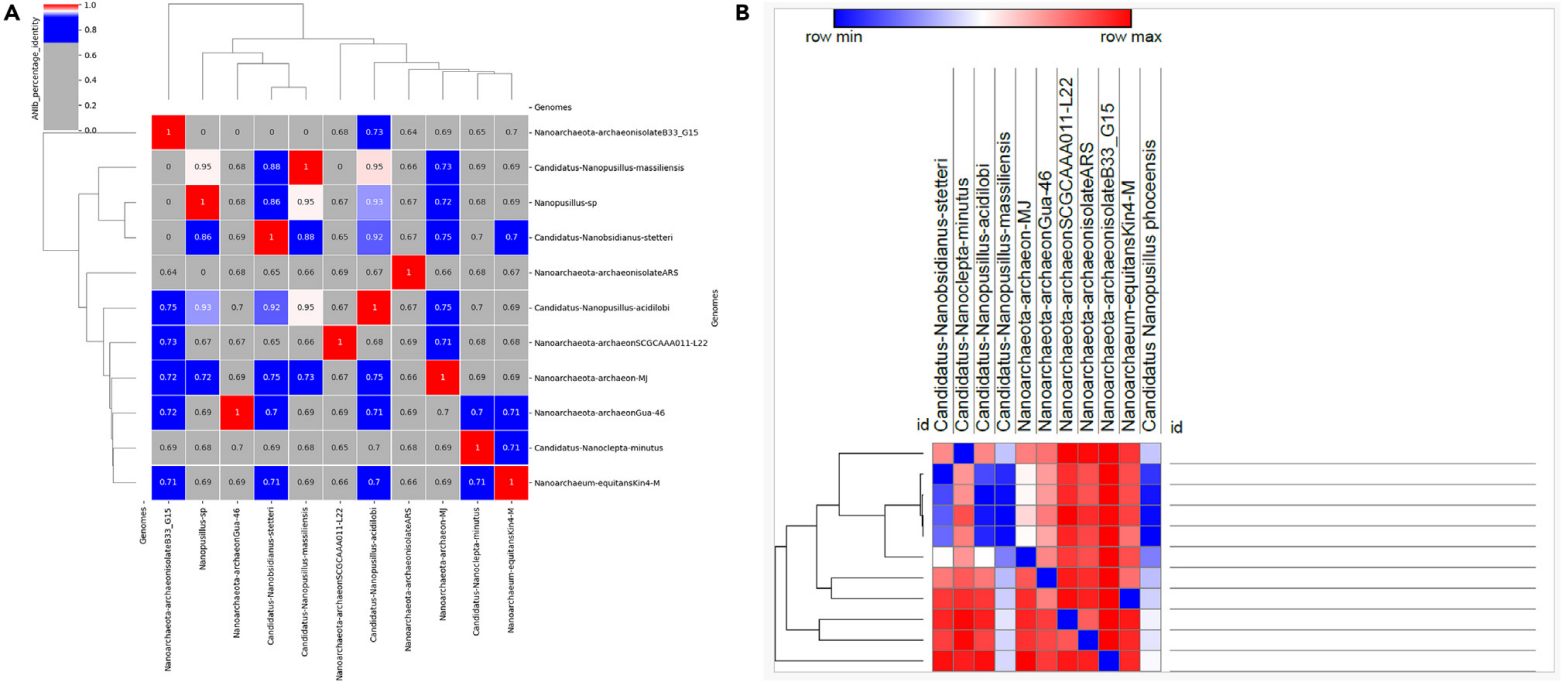
Genomics comparative of *Ca*. Nanopusillus foceencis (A) OrthoANI heatmap. (B) Single-nucleotide polymorphisms (SNPs) heatmaps.

**Figure 4:**
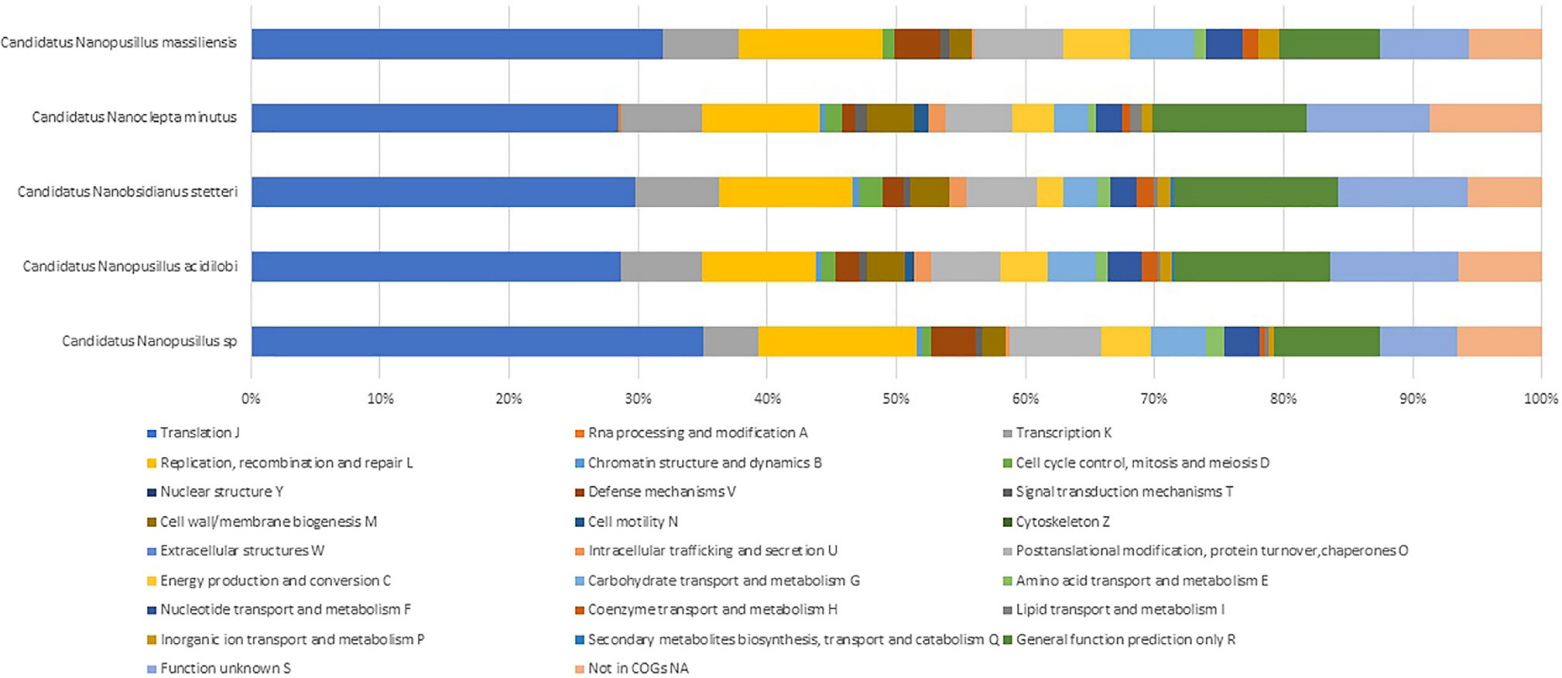
Number of genes associated with the 25 general COG functional categories

**Figure 5:**
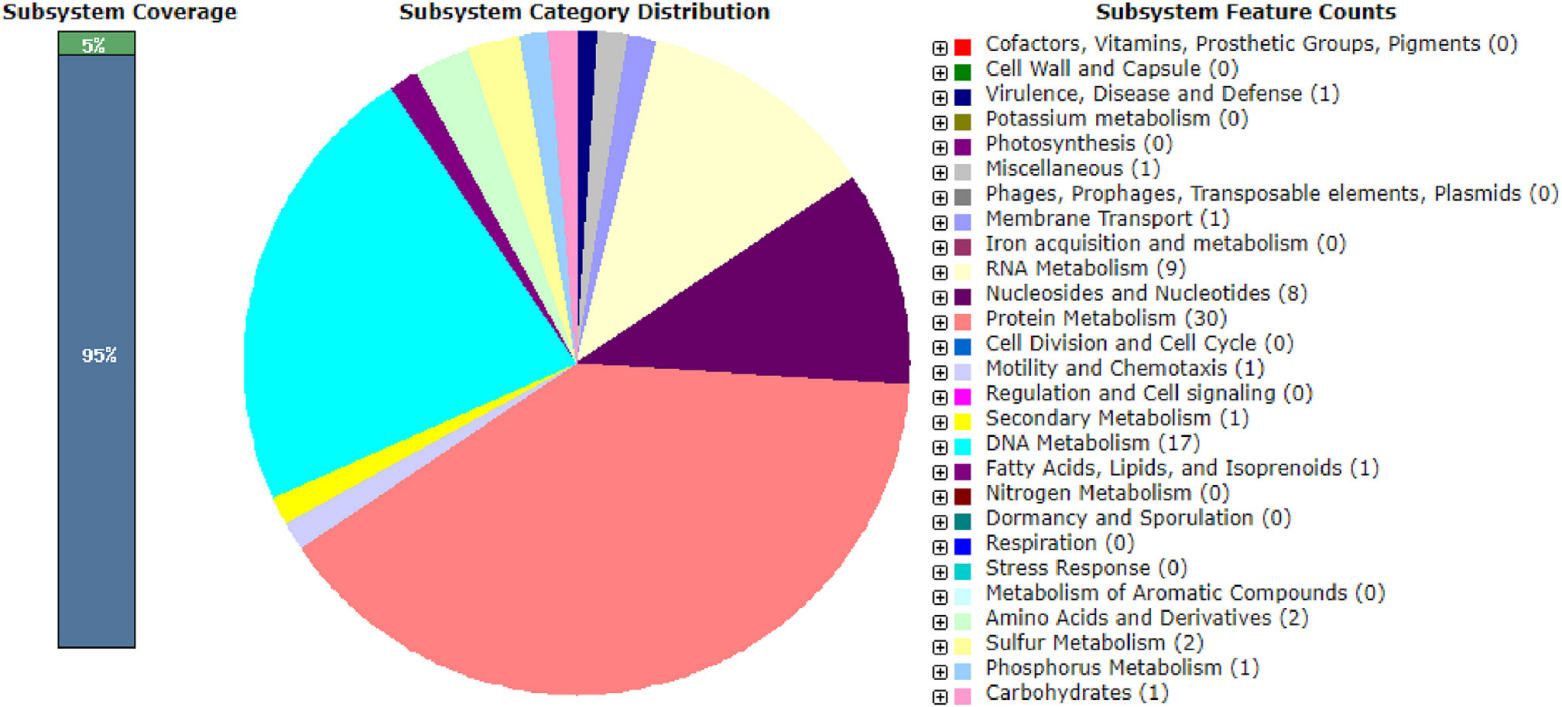
Phylogenetic tree based on pangenome highlighting the position of *Candidatus* Nanopusillus phoceensis compared to other Nanoarchaea taxa

**Figure 6:**
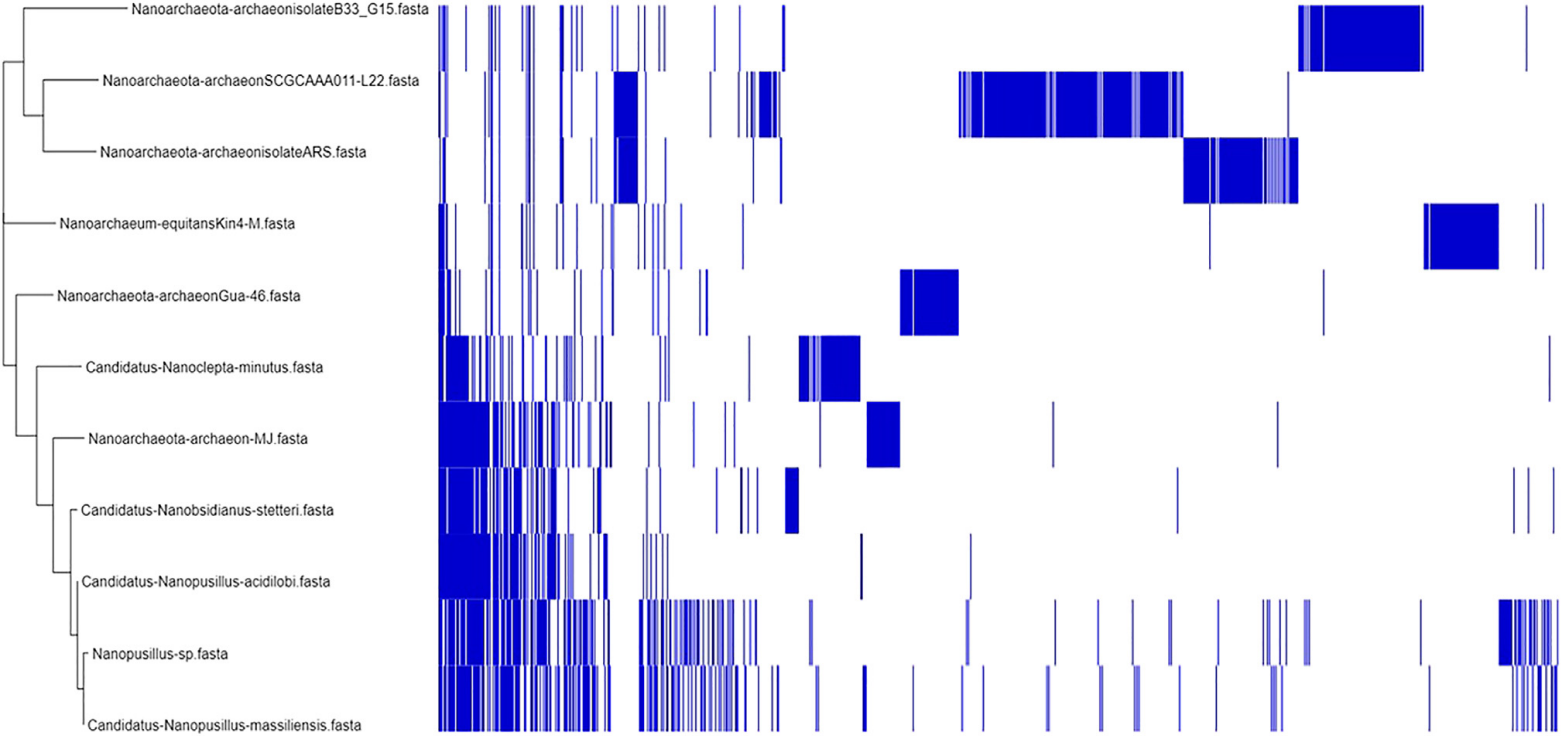
Phylogenetic tree based on pangenome highlighting the position of *Candidatus* Nanopusillus phoceensis compared to other Nanoarchaea taxa

**Figure 7:**
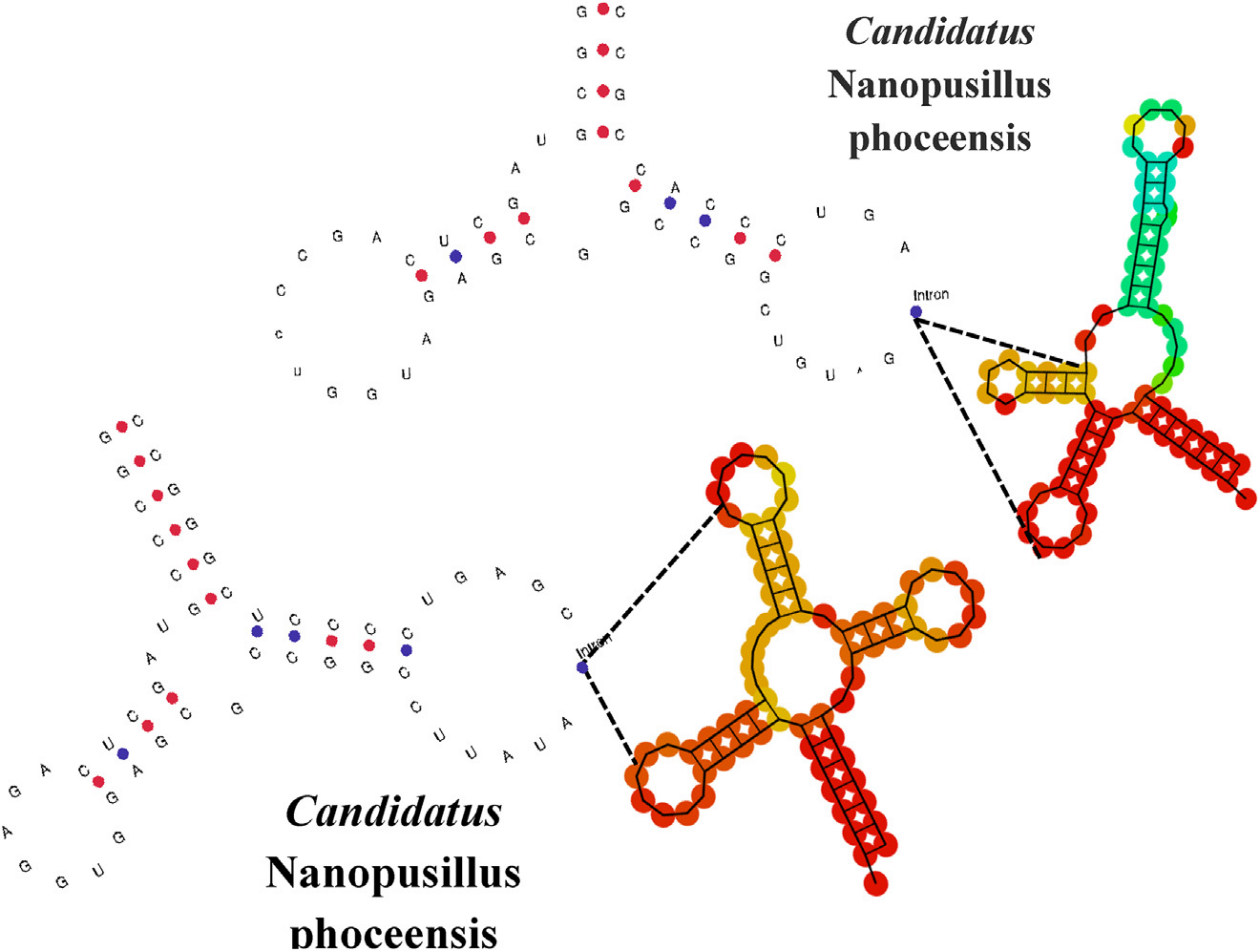
Two-dimensional representation of tRNAs with intronic sequences detected in *Candidatus* Nanopusillus phoceensis

**Figure 8:**
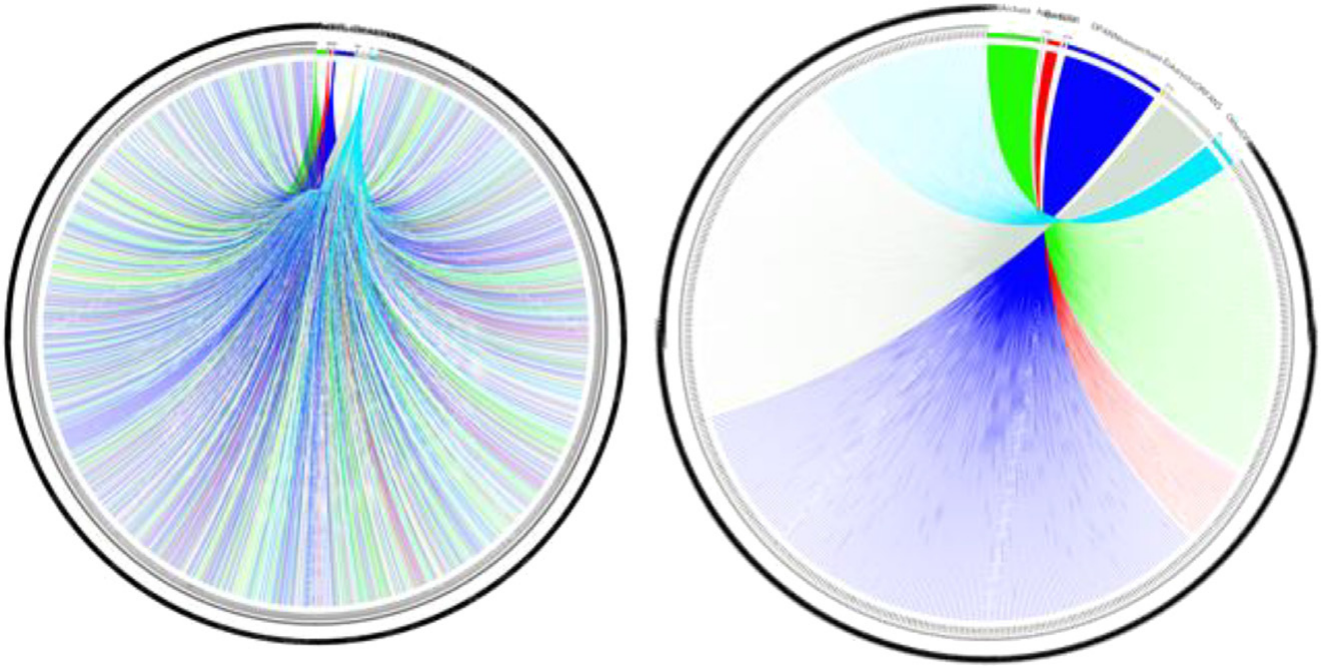
Rhizome representation of *Candidatus* Nanopusillus phoceensis

The Illumina and Nanopore sequencing yielded 7,516,594 bp which, mapped against the *N. massiliensis* reference genome, yielded a 607,775-bp contig, exhibiting a gap ratio of 2.4% representative of a draft genomic sequence named “*Candidatus* Nanopusillus phoceensis” (*Ca.* Nanopusillus phoceensis) with 24.5% G + C content. The OrthoANI analysis revealed a remarkably high similarity with a maximum value of 95.34% between *Ca*. Nanopusillus phoceensis and *N. massiliensis*. Additionally, there was a substantial 95% OrthoANI value between *Ca*. Nanopusillus phoceensis and *Ca*. Nanopusillus acidilobi. In contrast, the OrthoANI values dropped to less than 90% when comparing the new species with other nanoarchaea species (Figure 3A), which exhibited a maximum digital DNA-DNA hybridization (dDDH) value of 61.3 (confidence interval = [66.4–72.2]) (Table 1), indicating a new species. The *Ca.* Nanopusillus phoceensis genome encoded 30 tRNAs, 3 rRNA genes, and 1,403 coding DNA sequences, of which 719 were assigned to clusters of orthologous groups (COGs) (Figure 4 and Table S2 in the supplementary material). Of the 55 genes coding for enzymes, 30/55 (54.5%) encoded for enzymes involved in protein metabolism, 17/55 (30.9%) in DNA metabolism, and 9/55 (16.4%) in RNA metabolism (Figure 5). The pangenome of 11 nanoarchaea genomes including the one of *Ca.* Nanopusillus phoceensis incorporated 5,840 genes, including 4,635 cloud genes and 1,201 shell genes; these genomes shared four common genes: group_657, rplW, ruvB_2, and group_884 coding for 50S ribosomal protein L23; Holliday junction ATP-dependent DNA helicase; 50S ribosomal protein L18; and 30S ribosomal protein S19 (percentage basic local alignment search tool protein [BLASTp] identity = 50%) (Figure 6). Heatmap illustrated that the number of single-nucleotide polymorphisms (SNPs) found between the Nanoarchaea genomes studied here varied from six between *Ca.* Nanopusillus phoceensis and *N. massiliensis* to 463 with Nanoarchaeota-archaeon isolate B33_G15 (Figures 3B, and Table 2). We identified two tRNAs per genome that contained an intronic sequence, Ile TAT and Tyr GTA (Figure 7). A mosaic representation of the rhizome indicated that 652/1,642 (39.7%) nanoarchaea genes overlapped with DPANN (Diapherotrites, Parvarchaeota, Aenigmarchaeota, Nanoarchaeota, Nanohaloarchaeota)-nanoarchaea, 193/1,642 (11.75%) with other members of the DPANN superphylum, 320/1,642 (19.48%) with archaea, 78/1,642 (4.75%) with bacteria, 39/1,642 with the Asgard (0.54%), 6/1,642 with eukaryotes (0.36%), and 3/1,642 (0.18%) with Candidate phyla radiation, while 381/1,642 (23.2%) genes were orphans (Figure 8).

**Table 1:** Detailed table of digital DDH with values of 11 Nanoarchaea genomes

Query strain	Subject strain	dDDH (d4, in %)	C.I. (d4, in %)
*Nanopusillus acidilobi*	*Nanopusillus massiliensis*	62.2	[59.3–65.0]
*Nanopusillus massiliensis*	*Candidatus* Nanopusillus phoceensis '	61.3	[58.5–64.1]
*Nanopusillus acidilobi*	*Candidatus* Nanopusillus phoceensis '	50.0	[47.4–52.7]
Nanoarchaeota archaeonGua-46	*Nanoarchaeum equitans* Kin4-M	44.9	[42.3–47.5]
*Candidatus* Nanobsidianu stetteri	*Nanopusillus acidilobi*	44.9	[42.3–47.4]
*Candidatus* Nanobsidianus stetteri	Nanopusillus massiliensis	34.4	[32.0–36.9]
*Nanoclepta minutus*	Nanoarchaeota archaeonGua-46	32.9	[30.5–35.4]
*Candidatus* Nanobsidianus stetteri	*Candidatus* Nanopusillus phoceensis	30.4	[28.0–32.9]
*Nanoclepta minutus*	*Nanoarchaeum equitans* Kin4-M	27.5	[25.1–30.0]
*Candidatus* Nanobsidianus stetteri	*Nanoclepta minutus*	21.1	[18.9–23.5]
*Nanopusillus acidilobi*	*Nanoarchaeum equitansKin4-M*	18.9	[16.7–21.3]
*Candidatus* Nanobsidianus stetteri	*Nanoarchaeum equitns*Kin4-M	18.6	[16.4–21.0]
*Nanopusillus acidilobi*	Nanoarchaeota archaeon-MJ	18.5	[16.3–20.9]
*Nanopusillus acidilobi*	Nanoarchaeota archaeonGua-46	18.3	[16.1–20.6]
*Candidatus* Nanobsidianus stetteri	Nanoarchaeota archaeon-MJ	17.6	[15.5–20.0]
*Nanopusillus massiliensis*	Nanoarchaeota archaeon-MJ	17.4	[15.3–19.8]
*Nanoclepta minutus*	Nanoarchaeota archaeon-MJ	16.6	[14.5–18.9]
Nanoarchaeota archaeon-MJ	Candidatus Nanopusillus phoceensis	16.2	[14.1–18.5]
*Nanopusillus massiliensis*	*Nanoarchaeum equitans*Kin4-M	16.0	[14.0–18.3]
*Nanoclepta minutus*	*Nanopusillus acidilobi*	15.9	[13.8–18.2]
*Nanoclepta minutus*	*Nanopusillus massiliensis*	13.8	[11.9–16.0]
*Nanopusillus massiliensis*	Nanoarchaeota archaeonGua-46	13.7	[11.7–15.9]
Nanoarchaeota archaeonGua-46	*Candidatus* Nanopusillus phoceensis	13.5	[11.6–15.7]
Nanoarchaeota archaeonGua-46	Nanoarchaeota archaeonisolateARS	3.7	[2.8–4.8]
*Nanoclepta minutus*	Nanoarchaeota archaeonisolateB33 G15	3.7	[2.8–4.8]
Nanoarchaeota archaeonGua-46	Nanoarchaeota archaeon-MJ	3.7	[2.8–4.8]
*Nanopusillus acidilobi*	Nanoarchaeota archaeonisolateARS	3.7	[2.8–4.8]
Nanoarchaeota archaeonisolateARS	*Nanoarchaeum equitansKin4-M*	3.7	[2.8–4.8]
*Nanopusillus acidilobi*	Nanoarchaeota archaeonisolateB33 G15	3.7	[2.8–4.8]
*Candidatus* Nanobsidianus stetteri	Nanoarchaeota archaeonisolateB33 G15	3.7	[2.8–4.8]
*Nanoclepta minutus*	*Candidatus* Nanopusillus phoceensis	3.7	[2.8–4.8]
Candidatus Nanobsidianus stetteri	Nanoarchaeota archaeonisolateARS	3.7	[2.8–4.8]
*Nanopusillus massiliensis*	Nanoarchaeota archaeonisolateB33 G15	3.7	[2.8–4.8]
Nanoarchaeota archaeonisolateB33 G15	Nanoarchaeum equitansKin4-M	3.7	[2.8–4.8]
Nanoarchaeota archaeonisolateB33 G15	Nanoarchaeota archaeon-MJ	3.7	[2.8–4.8]
Nanoarchaeota archaeonisolateARS	*Candidatus* Nanopusillus phoceensis	3.7	[2.8–4.8]
*Nanoarchaeum equitans*Kin4-M	*Candidatus* Nanopusillus phoceensis	3.7	[2.8–4.8]
*Candidatus* Nanobsidianus stetteri	Nanoarchaeota archaeonGua-46	3.7	[2.8–4.8]
*Candidatus* Nanoclepta minutus	Nanoarchaeota archaeonisolateARS	3.7	[2.8–4.8]
Nanoarchaeota archaeonisolateB33 G15	*Candidatus* Nanopusillus phoceensis	3.7	[2.8–4.8]
Nanoarchaeota archaeonGua-46	Nanoarchaeota archaeonisolateB33 G15	3.7	[2.8–4.8]
Nanoarchaeota archaeonisolateARS	Nanoarchaeota archaeonisolateB33 G15	3.7	[2.8–4.8]
Nanoarchaeota archaeonisolateARS	Nanoarchaeota archaeon-MJ	3.7	[2.8–4.8]
*Nanopusillus massiliensis*	Nanoarchaeota archaeonisolateARS	3.7	[2.8–4.8]
Nanoarchaeota archaeon-MJ	*Nanoarchaeum equitans*Kin4-M	3.7	[2.8–4.8]

**Table 2:** The number of single-nucleotide polymorphisms (SNPs) found between the Nanoarchaea genomes

	*Candidatus Nanobsidianus stetteri*	*Candidatus Nanoclepta minutus*	*Candidatus Nanopusillus acidilobi*	*Candidatus Nanopusillus massiliensis*	*Nanoarchaeota archaeon-MJ*	*Nanoarchaeota archaeonGua-46*	*Nanoarchaeota archaeonSCGCA* *AA011-L22*	*Nanoarchaeota archaeonisolateARS*	*Nanoarchaeota archaeonisolate* *B33_G15*	*Nanoarchaeum-equitansKin4-M*	*Candidatus Nanopusillus phoceensis*
*Candidatus Nanobsidianus stetteri*	0	654	132	72	480	643	843	781	924	784	95
*Candidatus Nanoclepta minutus*	654	0	662	343	673	696	897	882	897	806	349
*Candidatus-Nanopusillus-acidilobi*	132	662	0	15	475	646	854	774	925	786	34
*Candidatus-Nanopusillus-massiliensis*	72	343	15	0	234	308	405	372	401	353	6
*Nanoarchaeota archaeon-MJ*	480	673	475	234	0	700	845	805	950	805	235
*Nanoarchaeota archaeonGua-46*	643	696	646	308	700	0	801	771	844	660	312
*Nanoarchaeota archaeonSCGC* *AAA011-L22*	843	897	854	405	845	801	0	740	907	864	431
*Nanoarchaeota archaeonisolateARS*	781	882	774	372	805	771	740	0	892	825	399
*Nanoarchaeota archaeonisolate* *B33_G15*	924	897	925	401	950	844	907	892	0	879	463
*Nanoarchaeum equitansKin4-M*	784	806	786	353	805	660	864	825	879	0	359
*Candidatus Nanopusillus phoceensis*	95	349	34	6	235	312	431	399	463	359	0

## Discussion

We discovered a new nanaorchaea species, which we named *Ca.* Nanopusillus phoceensis, from the human gut microbiota, as the result of adopting a polyphasic approach. First, molecular biology methods confirmed the presence of nanoarchaea DNA sequences in leftover human fecal samples, which were assigned to nano-organisms after congruent microscopy observations, in the presence of appropriate negative controls. Next-generation sequencing allowed us to obtain a genomic sequence related to the nanoarchaea genomic sequences. We succeeded in detecting, for the first time from digestive samples, *Ca.* Nanopusillus phoceensis with *M. smithii* as its host, as this was the only co-detected methanogen. To date, 11 nanoarchaea genomes are available online in the NCBI database (https://www.ncbi.nlm.nih.gov/), three of which are complete, including one of human origin (dental plaque). However, none of these genomes are from the human gut. Therefore, we compared the genome studied in this research with a set of nanoarchaea genomes available on NCBI. Due to the lack of data on the classification of nanaorchaea and the thresholds used to delineate one nanaorchaea species from another, we favored the thresholds used for the classification of “conventional” bacteria. *Ca.* Nanopusillus phoceensis shared four common genes with other nanoarchaea genomes, indicating both shared ancestry and divergence. Additionally, the analysis revealed varying degrees of genetic differentiation among nanoarchaea, as evidenced by the number of SNPs observed. Thus, we demonstrated that the OrthoANI and dDDH values calculated between the genome identified in the present study and those included were well below the recommended threshold values for delineating bacterial species (i.e., 95%–96% and 70%, respectively).^18^ All these data support the creation of a new nanaorchaea species from human fecal samples: *Ca.* Nanopusillus phoceensis. Regarding their origin, the presence of bacterial/eukaryotic sequences suggests the presence of an interaction between these nano-organisms in their shared niche. The mosaic structure of nanaorchaea in general gives them a unique characteristic, comparable to one another and different from other microbial domains. Microscopic observations showed a physical attachment between *Ca.* Nanopusillus phoceensis and *M. smithii*; accordingly *Ca.* Nanopusillus phoceensis encoded a flagellum component FlaI, which could play a role in the attachment, promoting metabolite exchanges. However, *Ca.* Nanopusillus phoceensis has conserved the genes encoding the mechanisms of replication, transcription, and translation as described for other nanoarchaea. We found a tRNA with intronic sequences in each genome, which has been recently described in nanoarchaea genomes, including *Nanoclepta minutus*^4^ and *Candidatus* Nanobsidianus stetteri,^5^ where two identical intronic sequences were identified that are absent in the host, suggesting that the intronic sequences were already present in the common ancestor of the nanoarchaea and were not transferred horizontally.^19^

The discovery of *Ca*. Nanopusillus phoceensis, the second nanoarchaea found in humans, represents a significant expansion of our knowledge of the repertoire of nanoarchaea in the human microbiota indicating that nanoarchaea are not specifically associated with *M. oralis*^6^ but most possibly to any member of the *Methanobrevibacter* genus at large. The polyphasic approach which we initially used for oral human samples could also be used for other microbiota, such as the human gut microbiota, as described here, and can be used in other samples from other microbiota for the discovery of new genomic sequences. This would help us better understand the physiological role of nanoarchaea as well as their dynamic relationship with other microorganisms.

Indeed, the discovery of new nanoarchaea in clinical microbiology is a significant development that expands our understanding of human microbiome networks. These nano-organisms have unveiled their presence in various human body sites (oral and gut microbiota), adding to the growing diversity of microorganisms within human mucosae-associated microbiota. Perhaps even more fascinating are the potential symbiotic relationships that nanoarchaea may form with other microorganisms within our bodies. These relationships offer glimpses into the complex interactions and coexistence of diverse microbes in the human microbiome. Furthermore, the implications of nanoarchaea’s presence in clinical samples for human health and disease are a subject of growing interest. Research into their role could yield insights into conditions associated with the human microbiome and possibly lead to innovative diagnostic or therapeutic applications. Beyond health, this discovery also enriches our knowledge of microbial ecosystems, both within the human body and in various environments, shedding light on the broader microbial ecology of our planet. Moreover, the genomic information gleaned from nanoarchaea opens exciting avenues for research in microbiology, genomics, and evolutionary biology, further fueling scientific advancement. In essence, the discovery of these new nanoarchaea is a testament to the rich and still largely uncharted microbial diversity on human microbiota.

Future studies in the field of nanoarchaea should focus on addressing several key aspects. Firstly, an expansion of the dataset to encompass nanoarchaeal genomes from diverse environments, including extreme ecosystems, can provide a more comprehensive understanding of their diversity and functional adaptations. Secondly, functional characterization of specific genes and proteins within nanoarchaea, coupled with metagenomic analyses, will help elucidate their ecological roles in various ecosystems, including the human microbiota. Additionally, efforts to isolate and cultivate nanoarchaea in the laboratory will facilitate in-depth investigations into their physiology and metabolism. Lastly, functional genomics techniques, such as transcriptomics and proteomics, should be employed to uncover the gene expression patterns and protein functions of nanoarchaea, shedding light on their contributions to microbial communities and their potential biotechnological applications. In this groundbreaking study, we have made a significant discovery by identifying a nanoarchaea species, Candidatus Naniopusillus phoceensis, originating from the digestive microbiota. This discovery represents a major milestone as it provides only the second genomic sequence of a nanoarchaea of human origin. Through our research, we have successfully validated a protocol that was previously developed in another study to detect nanoarchaea in clinical samples. This validation is highly encouraging and opens new possibilities for future studies aimed at expanding our knowledge of nanoarchaea associated with the human microbiota. The identification of new nanoarchaea species and the acquisition of additional genomic sequences hold immense potential in advancing our understanding of their diversity, phenotypic traits, and phylogenetic characteristics. Moreover, this knowledge will shed light on the physiological roles that nanoarchaea play within the ecosystems they inhabit.

As we delve deeper into this uncharted territory of nanoarchaea research, we anticipate uncovering invaluable insights into their ecological significance and their interactions within the human microbiota. This study marks a crucial step forward in the field of microbiology and has the potential to contribute significantly to our knowledge of the microbial world and its impact on human health.

### Limitations of the study

Our study has several limitations. The study was conducted on a relatively small number of stool specimens (110), and a larger sample size could provide a more comprehensive understanding of the prevalence and diversity of nanoarchaea in the human microbiota. Futher, Nanoarchaea are notoriously difficult to cultivate, and the study relied on molecular and microscopic methods. Cultivation techniques could provide more detailed insights into the characteristics and behavior of *Candidatus* Nanopusillus phoceensis. While the study observed a close association between Candidatus Nanopusillus phoceensis and Methanobrevibacter smithii, the functional aspects of this relationship and its implications for host health remain speculative and warrant further investigation.

## STAR★Methods

### Key resources table

**Table:** 

REAGENT or RESOURCE	SOURCE	IDENTIFIER
**Other**		

Extraction buffer	Qiagen	ID: 1014636
Fastprep Bio 101 apparatus	Qbiogene	SKU:116004500
Proteinase K	Qiagen	ID:P103B-20MG
Amplitaq Gold	Thermofisher	4398813
Glutaraldehyde solution	Sigma-Aldrich	111-30-8
Phosphotungstic acid	Sigma-Aldrich	12501-23-4
Nextera XT DNA	Illumina	FC-131-1096
AMPure XP beads	Beckman Coulter	A63882
Qubit assay kit	Thermofisher	Q32851

**Oligonucleotides**		

5′- TGAAAGCAAAGGGATTTTATTCA-3′	Hassani et al., ^6^	Forward primer
5′-TTGCATGTGGAACAATACCAG-3′	Hassani et al., ^6^	Reverse primer
5′-GTGCTCCCCCGCCAATTCCT-3′	Hassani et al., ^6^	Arch915 probe Alexa 647
5′-CCCTCTGGCCCACTGCT-3′	Wurch et al.,^3^	Nano Alexa 488

### Resource availability

#### Lead contact

Further information and requests for resources and reagents should be directed to and will be fulfilled by the lead contact, Ghiles GRINE (grineghiles@gmail.com).

#### Materials availability

This study did not generate new unique reagents.

#### Data and code availability

*Candidatus* Nanopusillus Phoceensis genome accession:

ASSEMBLY_NAME | ASSEMBLY_ACC | STUDY_ID | SAMPLE_ID | CONTIG_ACC | SCAFFOLD_ACC | CHROMOSOME_ACCQ6268 | GCA_920984865 | PRJEB46774 | ERS8452987 | CAKLBW010000001-CAKLBW010000593 | OV100765-OV100765 |

This study does not report original new code. Any additional information required to reanalyze the data reported in this paper is available from the lead contact upon request.

### Experimental model and study participant details

#### Sample collection

A series of 110 leftover stool samples which were previously collected at the Institut Hospitalo-Universitaire Méditerranée Infection for the study the prevalence of methanogens and nanoarchaea, were stored at 4°C until use. According to our experience, storing methanogens at −80°C can be detrimental to their viability, whereas storage at 4°C better preserves methanogen viability. At −80°C, the formation of ice crystals can lead to damage to the methanogen cell walls. Only one stool sample was collected per person. This study only involved anonymous samples that were not obtained specifically for the present study but rather were clinical samples which were left over after required diagnostic staging. Patients had been informed of the possible use of leftover samples for research purposes and retained their right to refuse approval at any time. According to the Jardé Law (Law No. 2012-300 of 5 March 2012 and Decree No. 2016-1537 of 16 November 2016 published in the Journal Officiel de la République Française), this study did not involve any specific sample collection or use of patients medical/personal data. As a result, neither institutional ethical approval nor individual patient consent were required for this study.

### Method details

#### PCR based detection

A 0.2 g aliquot of each leftover stool sample suspended in 500 μL of G2 buffer (QIAGEN, Hilden, Germany) in an Eppendorf tube (Fisher Scientific, Illkirch, France) was mixed with 0.3 g of acid washed glass beads ≤106 mm (Sigma, Saint-Quentin Fallavier, France) and shaken to obtain mechanical lysis in a FastPrep BIO 101 apparatus (Qbiogene, Strasbourg, France) at level 6.5 (full speed) for 90 s. The supernatant was incubated at 100°C for 10 min and a 180 μL-volume was further incubated with 20 μL of proteinase K (QIAGEN) at 56°C overnight. Total DNA was extracted with the EZ1 Advanced XL extraction kit (QIAGEN), eluted with 100 μL of elution buffer and stored at −20°C until use. A mock extraction performed with 200 μL of sterile water was used as a negative control for each batch of DNA extraction. Extracted DNA and the control were incorporated into PCR-sequencing for the detection of nanoarchaea using a specific primer pair targeting the broad range archaeal 30S SSU L12 gene (forward primer: 5′-TGAAAGCAAAGGGATTTTATTCA-3’; reverse primer 5′-TTGCATGTGGAACAATACCAG-3′), incorporated into a 50 μL volume containing 25 μL Amplitaq Gold (ThermoFisher Scientific), 2 μL of each primer (10 p.m.) (Eurogentec, Seraing, Belgium), 16 μL of DNase/RNase-free distilled water (Gibco, Cergy-Pontoise, France), and 5 μL of extracted DNA. The reaction mixture was then subjected to a 40-cycle PCR program comprising a 30-s denaturation step at 95°C, followed by 45-s hybridisation at 60°C and a 1-min elongation at 72°C. Each amplification program started with a 15-min denaturation step at 95°C and ended with a final 5-min elongation step at 72°C. PCR products were sequenced as previously described.^20^ In parallel, samples and negative controls were assayed for the presence of methanogens by PCR-sequencing, targeting the broad range archaeal 16S rRNA gene, as previously reported.^20^

#### Microscopic observations of nanoarchaea

Eighteen Nanoarchaea positive PCR-sequencing sample were analyzed according to this method; 0.2 g of leftover stool samples, suspended in 1 mL of sterile Phosphate Buffered Saline (PBS, ThermoFisher Scientific) was centrifuged at 2,500 g for 10 min. The suspension was diluted 1:100 with PBS and fixed with a 2.5% glutaraldehyde solution (Sigma) and cyto-centrifuged onto a cytospin slide contrasted with 1% aqueous phosphotungstic acid PTA solution (Sigma-Aldrich, St. Louis, MO, USA) (pH = 7) for 2 min. Images were acquired using the Hitachi TM4000 Plus tabletop SEM (Hitachi, Tokyo, Japan), using the Electron Backscatter (BSE) as a detector, to observe the structure of the nanoarchaea. The accelerating voltage was 15 kV and magnifications varied from 250× to 7,000 X. All samples were acquired using the same acquisition parameters regarding magnification, intensity, and voltage mode. Also, fluorescent *in situ* hybridisation (FISH) incorporated the archaea-specific Arch915 probe Alexa 647 (5′-GTGCTCCCCCGCCAATTCCT-3′)^6^ and the nanoarchaeota 16S rRNA gene probe 515mcR2 probe Alexa 488 (5′-CCCTCTGGCCCACTGCT-3′), as previously described.^3^

#### Next-generation sequencing and sequence analyses

Ten stool sample which were nanoaarchaea PCR-sequencing positive have been sequenced following the method below: DNA extracted as above was sequenced using the MiSeq instrument and the Nextra XT DNA sample prep kit and paired-end strategy (Illumina Inc., San Diego, CA, USA). The tagmentation step fragmented and tagged each extracted DNA to prepare the paired-end library. Twelve cycles of limited PCR amplification were performed to complete tag adapters and to introduce dual-index barcodes. DNA was then purified on AMPure XP beads (Beckman Coulter Inc., Fullerton, CA, USA). In addition, in line with the Nextera XT protocol, all libraries were normalised on specific beads and pooled for DNA sequencing. The pooled single strand library was loaded onto the reagent cartridge and then onto the instrument along with the flow cell. Automated cluster generation and paired-end sequencing with dual index reads were performed in a single 39-h run in 2 × 250-bp to attain a substantial sequencing depth. In addition, the Oxford Nanopore method was performed for 1D genomic DNA sequencing on the GridION device, using the SQK-LSK109 Kit (Oxford Nanopore, Oxford, U.K). A library was constructed from 1 μg genomic DNA without fragmentation and end-repair. Adapters were ligated to both ends of the genomic DNA. After purification on AMPure XP beads (Beckman Coulter, Inc.), the library was quantified by a Qubit assay with the high-sensitivity kit (Life Technologies, Carlsbad, CA, USA). After detection of active pores for sequencing, the WIMP (What’s In My Pot) workflow was chosen for live bioinformatic analyses. Quality of each read was checked by FastQC and trimmed using Trimmomatic version 0.36.6^21^ and all the reads corresponding to one single given sample were merged. Each group of reads was mapped against the reference nanoarchaea genome (*N. massiliensis*, available in NCBI under accession number CAKLBW00000000) using CLC Genomics Workbench v.7 with default parameters except for the length fraction (reduced to 0.3) and similarity fraction (reduced to 0.5). Mapped reads were assembled using SPAdes software, version 3.13.0 using the default options, and keeping only contigs >400 bp. Each contig was BLASTn against the nr database to keep contigs matching with Nanoarchaea spp. sequences. Such selected fasta sequences were mapped against the *N. massiliensis* genome using the above-mentioned criteria to generate an almost complete nanoarchaea genome, with no contamination. The new genome was deposited in GenBank. Hypothetical proteins, coding sequence (CDS) and rRNA were predicted using Prokka.^22^ To determine the mosaicism and evolutionary history of each genome, we constructed a representative rhizome featuring genetic exchanges between the newly sequenced nanoarchaea genome and other organisms: a BLASTp for each CDS was performed against the NCBI protein database and any protein-coding gene which did not match with any sequence was considered an ORFan. The remaining best HITs were selected based on the following criteria: minimum identity and coverage of 20% and 30% respectively and maximum e-value of 0.001, as previously described.^23^ Rhizome representations were then constructed using the Circos software.^24^ For taxonomic characterisation, we selected for comparison all nanoarchaea genome sequences available in the NCBI database. Genomic similarity was estimated using OrthoANI software and the Genome-to-Genome Distance Calculator Web Service to calculate the digital dDDH value with confidence intervals according to recommended parameters, as a previously described.^25^ Finally, tRNA genes were predicted by tRNA SCAN SE,^26^ using the default option and all available sequence sources.
