# Supplementary material for: The discovery of Candidatus Nanopusillus phoceensis sheds light on the diversity of the microbiota nanoarchaea
Source: iScience. 2024 Mar 11;27(4):109488. doi: 10.1016/j.isci.2024.109488 (PMC11001627; doi:10.1016/j.isci.2024.109488)
Supplement: Document S1. Tables S1 and S2 [file mmc1.pdf]

iScience, Volume *27*

Supplemental information

The discovery of *Candidatus* Nanopusillus phoceensis sheds light on the diversity of the microbiota nanoarchaea

Yasmine Hassani, Gerard Aboudharam, Michel Drancourt, and Ghiles Grine

SUPPLEMENTAL INFORMATION TITLES AND LEGENDS

Table S1: All the results of PCR-sequencing-based investigation of methanogens/nanoarchaea.

Stools	Methanogens PCR-Sequencing	Nanoarchaea PCR-sequencing
**1**	*M. smithii*	*N. massiliensis*
**2**	*M. smithii*	Negative
**3**	*M. smithii*	*N. massiliensis*
**4**	*M. smithii*	Negative
**5**	*M. smithii*	Negative
**6**	*M. smithii*	Negative
**7**	*M. smithii*	Negative
**8**	Negative	Negative
**9**	*M. smithii*	Negative
**10**	*Methanomassiliicoccaceae archaeon DOK*	Negative
**11**	*M. smithii*	Negative
**12**	*M. smithii*	Negative
**13**	*M. smithii*	*N. massiliensis*
**14**	*M. smithii*	Negative
**15**	*M. smithii*	Negative
**16**	*Methanomassiliicoccaceae archaeon DOK*	Negative
**17**	*M. smithii*	Negative
**18**	*M. smithii*	Negative
**19**	*M. smithii*	*N. acidilobi*
**20**	*M. smithii*	Negative
**21**	*M. smithii*	Negative
**22**	*M. smithii*	*N. massiliensis*
**23**	*M. smithii*	*N. acidilobi*
**24**	*M. smithii*	Negative
**25**	*M. smithii*	Negative
**26**	*M. smithii*	Negative

**27**	*M. smithii*	Negative
**28**	*M. smithii*	Negative
**29**	*M. smithii*	Negative
**30**	Negative	Negative
**31**	Negative	Negative
**32**	*M. oralis, strain VD9*	*N. massiliensis*
**33**	*M. smithii*	Negative
**34**	*M. smithii*	Negative
**35**	*M. smithii*	Negative
**36**	*M. smithii*	Negative
**37**	Negative	Negative
**38**	Negative	Negative
**39**	*M. smithii*	*N. acidilobi*
**40**	Negative	Negative
**41**	*M. smithii*	*N. acidilobi*
**42**	Negative	Negative
**43**	*M. smithii*	Negative
**44**	*M. smithii*	Negative
**45**	*M. smithii*	Negative
**46**	Negative	Negative
**47**	*M. smithii*	*N. massiliensis*
**48**	Negative	Negative
**49**	*Methanobrevibacter* sp. AbM23	Negative
**50**	Negative	Negative
**51**	Negative	Negative
**52**	*M. smithii*	*N. acidilobi*
**53**	*M. smithii*	Negative
**54**	*M. smithii*	Negative
**55**	*M. smithii*	Negative
**56**	*Candidatus Methanomassiliicoccus intestinalis*	Negative

**57**	Negative	Negative
**58**	Negative	Negative
**59**	*M. smithii*	Negative
**60**	*M. smithii*	Negative
**61**	*M. smithii*	Negative
**62**	*M. smithii*	*N. massiliensis*
**63**	Negative	Negative
**64**	*M. smithii*	*N. acidilobi*
**65**	Negative	Negative
**66**	*M. smithii*	*N. massiliensis*
**67**	*M. smithii*	*N. acidilobi*
**68**	Negative	Negative
**69**	Negative	Negative
**70**	*M. smithii*	Negative
**71**	*M. smithii*	Negative
**72**	*M. smithii*	Negative
**73**	*M. smithii*	Negative
**74**	*M. smithii*	Negative
**75**	*M. smithii*	Negative
**76**	*M. smithii*	Negative
**77**	*M. smithii*	Negative
**78**	*M. smithii*	Negative
**79**	*M. smithii*	Negative
**80**	*M. smithii*	Negative
**81**	*M. smithii*	Negative
**82**	*M. smithii*	Negative
**83**	*M. smithii*	Negative
**84**	Negative	Negative
**85**	*M. smithii*	Negative
**86**	*M. smithii*	Negative

**87**	*M. smithii*	Negative
**88**	*M. smithii*	Negative
**89**	*M. smithii*	Negative
**90**	*M. smithii*	Negative
**91**	*M. smithii*	Negative
**92**	*M. smithii*	Negative
**93**	*M. smithii*	Negative
**94**	*M. smithii*	Negative
**95**	*M. smithii*	Negative
**96**	*M. smithii*	Negative
**97**	*M. smithii*	*N. acidilobi*
**98**	*M. smithii*	Negative
**99**	*M. smithii*	Negative
**100**	*M. smithii*	Negative
**101**	*M. smithii*	Negative
**102**	*M. smithii*	Negative
**103**	*M. smithii*	Negative
**104**	*M. smithii*	*N. massiliensis*
**105**	*M. smithii*	Negative
**106**	*M. smithii*	Negative
**107**	*M. smithii*	Negative
**108**	*M. smithii*	Negative
**109**	*M. smithii*	Negative
**110**	*M. smithii*	Negative

Table S2: Numbers of genes of Candidatus Nanopusillus phoceensis associated with general COG functional categories.

protein function	COGs	*Candidatus* Nanopusillus phoceensis	*Nanopusillus acidilobi*	*Candidatus* Nanobsidianus stetteri	*Candidatus* Nanoclepta minutus	*Nanopusillus massiliensis*
Translation	J	199	128	115	131	135
Rna processing and modification	A	0	0	0	1	0
Transcription	K	24	28	25	29	25
Replication, recombination and repair	L	70	39	40	42	47
Chromatin structure and dynamics	B	2	2	2	2	0
Cell cycle control, mitosis and meiosis	D	4	5	7	6	4
Nuclear structure	Y	0	0	0	0	0
Defense mechanisms	V	20	8	6	5	15
Signal transduction mechanisms	T	3	3	2	4	3
Cell wall/membrane biogenesis	M	10	13	12	17	7
Cell motility	N	0	3	0	5	0
Cytoskeleton	Z	0	0	0	0	0
Extracellular structures	W	0	0	0	0	0
Intracellular trafficking and secretion	U	2	6	5	6	1
Posttanslational modification, protein turnover,chaperones	O	40	24	21	24	29
Energy production and conversion	C	22	16	8	15	22
Carbohydrate transport and metabolism	G	24	17	10	12	21

**Amino acid transport and metabolism**	E	8	4	4	3	4
**Nucleotide transport and metabolism**	F	16	12	8	9	12
**Coenzyme transport and metabolism**	H	2	5	5	3	5
**Lipid transport and metabolism**	I	2	1	1	4	0
**Inorganic ion transport and metabolism**	P	2	4	4	4	7
**Secondary metabolites biosynthesis, transport and catabolism**	Q	0	1	1	0	0
**General function prediction only**	R	47	54	49	55	33
**Function unknown**	S	34	44	39	44	29
**Not in COGs**	NA	37	29	22	40	24
